# Next‐generation sequence‐based preimplantation genetic testing for monogenic disease resulting from maternal mosaicism

**DOI:** 10.1002/mgg3.1662

**Published:** 2021-05-04

**Authors:** Xiao Hu, Wen‐Bin He, Shuo‐Ping Zhang, Ke‐Li Luo, Fei Gong, Jing Dai, Yi Zhang, Zhen‐Xing Wan, Wen Li, Shi‐Min Yuan, Yue‐Qiu Tan, Guang‐Xiu Lu, Ge Lin, Juan Du

**Affiliations:** ^1^ Reproductive and Genetic Hospital of CITIC‐Xiangya Changsha China; ^2^ Institute of Reproduction and Stem Cell Engineering School of Basic Medical Science Central South University Changsha China; ^3^ Key Laboratory of Stem Cell and Reproduction Engineering Ministry of Health Changsha China; ^4^ National Engineering and Research Center of Human Stem Cell Changsha China

**Keywords:** maternal mosaicism, monogenic disease, next‐generation sequencing, preimplantation genetic testing

## Abstract

**Background:**

Mosaicism poses challenges for genetic counseling and preimplantation genetic testing for monogenic disorders (PGT‐M). NGS‐based PGT‐M has been extensively used to prevent the transmission of monogenic defects, but it has not been evaluated in the application of PGT‐M resulting from mosaicism.

**Methods:**

Four women suspected of mosaicism were confirmed by ultra‐deep sequencing. Blastocyst trophectoderm cells and polar bodies were collected for whole genome amplification, followed by pathogenic variants detection and haplotype analysis based on NGS. The embryos free of the monogenic disorders were transplantable.

**Results:**

Ultra‐deep sequencing confirmed that the four women harbored somatic mosaic variants, with the proportion of variant cells at 1.12%, 9.0%, 27.60%, and 91.03%, respectively. A total of 25 blastocysts were biopsied and detected during four PGT cycles and 5 polar bodies were involved in one cycle additionally. For each couple, a wild‐type embryo was successfully transplanted and confirmed by prenatal diagnosis, resulting in the birth of four healthy infants.

**Conclusions:**

Mosaic variants could be effectively evaluated via ultra‐deep sequencing, and could be prevented the transmission by PGT. Our work suggested that an NGS‐based PGT approach, involving pathogenic variants detection combined with haplotype analysis, is crucial for accurate PGT‐M with mosaicism.

## Graphical abstract

NGS‐based PGT is an effective strategy for the detection of monogenic diseases resulting from mosaicism. Furthermore, NGS‐based PGT combined with PB genetic analysis can improve the accuracy of PGT for monogenic diseases due to maternal mosaicism. This approach will assist clinicians to undertake a more objective assessment of disease recurrence risk for mosaic individuals and obtain an accurate diagnosis of the genotype of embryos for reproductive interventions.

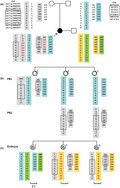

## 1 INTRODUCTION

Mosaicism refers to the presence of two or more genotypically distinct cell populations in an individual due to post‐zygotic *de novo* sequence variants and may include somatic, germline, and gonosomal mosaicism (a combination of somatic mosaicism and germline mosaicism) (Biesecker & Spinner, 2013; Campbell et al., 2015). Somatic mosaicism typically manifests distinct clinical phenotypes depending on the distribution and extent of the variants (Malcov et al., 2010; Tarilonte et al., 2018). Although germline mosaicism does not lead to clinical phenotypes, it may be passed on to future generations and result in disorders (Altarescu et al., 2012; Patel et al., 2018; Viart et al., 2017). Mosaic genetic variants display a "non‐Mendelian" inheritance pattern and thus pose challenges in genetic counseling and result in an underestimated or overestimated recurrence risk in offspring (Wright et al., 2019). Therefore, it is essential to develop accurate tools to identify mosaicism.

A range of molecular biology techniques with varied sensitivities is applied in the detection of mosaicism for single nucleotide variants or small copy number variants,including Sanger sequencing, multiplex ligation‐dependent probe amplification (MLPA), various PCR techniques, and next‐generation sequencing (NGS) (Gajecka, 2016). Sanger sequencing and MLPA, two classical techniques applied in diagnostic laboratories, exhibit low sensitivity in detecting low‐grade mosaicism, in which mosaic variants are usually disregarded as a background signal if the altered allele is at a low level (Jamuar et al., 2014; Summerer et al., 2019). Although allele‐specific PCR is more sensitive than Sanger sequencing, it has the disadvantage of low throughput and can only be used to amplify the altered allele (Ihle et al., 2014). NGS identifies very low‐abundance variants with high sensitivity because it performs massive, parallel analysis of thousands of DNA fragments (Miyatake et al., 2014; Summerer et al., 2019). Thus, NGS is deemed to be the first choice for the identification of mosaicism.

The detection of mosaicism provides a basis for precise reproductive interventions. Preimplantation genetic testing (PGT) is a powerful tool for patients to prevent the transmission of genetic aberrations to the next generation. There are three popular technologies applied in PGT for monogenic diseases (PGT‐M), including multiplex fluorescent PCR, karyomapping (Handyside et al., 2010), and NGS. Although multiplex fluorescent PCR and karyomapping have been used in PGT‐M for many years, these methods have few disadvantages. Multiplex fluorescent PCR is a time‐consuming and labor‐intensive technique (Khosravi et al., 2016), and karyomapping has a potential risk of misdiagnosis due to lack of direct pathogenic variants detection (Konstantinidis et al., 2015). In contrast, NGS‐based PGT‐M, which combines pathogenic variants detection with haplotype analysis, has the advantage of high efficiency and low labor cost and is gaining prominence. Herein, we performed ultra‐deep sequencing to confirm mosaic variants in the subjects. NGS‐based PGT was subsequently used to reduce the recurrence risk of monogenic diseases in these families, resulting in healthy live births.

## 2 MATERIALS

### 2.1 Patients

The couples were recruited from the Reproductive and Genetic Hospital of CITIC Xiangya (Changsha, China) between December 2017 and May 2019 according to previously described criteria. Briefly: (a) one partner with or without clinical symptoms in the couple carried a *de novo* pathogenic sequence variant (Biesecker & Spinner, 2013; Naja et al., 2016; Steffann et al., 2014), (b) the asymptomatic parents, who had one or more children affected with the same disorder, did not possess the genomic alterations carried by the children as per Sanger sequencing (Altarescu et al., 2012; Rechitsky et al., 2011). The written informed consent were obtained from these couples.

### 2.2 Evaluation of mosaicism

Peripheral blood samples were collected from these couples and their relatives, and oral mucosa cells were collected, when available. Genomic DNA was extracted using the QIAamp DNA Blood Mini Kit (QIAGEN). The DNA samples of individuals suspected to carry mosaic variants were sent to Jiajian Medicine Tech., Ltd for ultra‐deep sequencing. Target variant sites were amplified using the Taq Ready Mix (Takara), followed by library preparation and sequencing on a NEXTSeq 500 system (Illumina). The proportion of mosaicism was calculated based on the depth of ultra‐deep sequencing (Biesecker & Spinner, 2013; Summerer et al., 2019).

### 2.3 Haplotype construction

Preliminary experiments on genomic DNA were conducted before PGT; the pathogenic variants and the linked single nucleotide polymorphism (SNP) of target genes were identified, and the haplotypes were constructed according to family history. The SNPs in a 2 Mb range on each side of the target gene with minor allele frequency >0.2 in the 1000 Genomes Project were selected. The number of SNPs was generally more than 200. In addition, the SNPs on the Y chromosome were also included in the case of X‐linked diseases. Primers were designed (https://www.ampliseq.com/), and the amplicon sizes were between 125 and 275 bp. The SNP haplotype construction was performed by Peking Jabrehoo Med Tech., Ltd. Briefly, multiplex PCR was performed to capture the coding region of the target gene and the relevant SNPs. Then, multiple samples were added with different label sequences (barcode), followed by library preparation by the Illumina standard process. Finally, sequencing was performed using the MiSeq Sequencing System (Illumina) with an average depth of over 100X. The sequencing data were analyzed by a software developed by Peking Jabrehoo Med Tech., Ltd. The reference genome was human GRCh37/hg19.

### 2.4 In vitro fertilization, biopsy, and vitrification

The couples underwent In vitro fertilization (IVF)/intracytoplasmic sperm injection (ICSI) treatment, biopsy, and embryo vitrification as previously reported (Tan et al., 2014). Briefly, the first polar body (PB) was removed 1–2 h after ICSI fertilization on the day of oocyte retrieval (day 0), and the second PB was removed using the same zona pellucida opening on day 1 (Levin et al., 2012). Blastocyst trophectoderm cells were biopsied on day 5 or day 6 after fertilization, depending on embryo development. Each biopsied sample was transferred to a separate 0.2 ml tube, and the blank culture medium and the last wash droplet of each biopsied sample were also collected to ensure the absence of contamination.

### 2.5 Genetic testing

Blastocyst trophectoderm cells, PBs, blank culture medium, and the last wash droplet of each biopsied sample were amplified by REPLI‐g Single Cell Kit (QIAGEN, Hilden, Germany) as per manufacturer's instructions. The amplified products were tested for mutated sites and linked SNPs at Peking Jabrehoo Med Tech., Ltdfor haplotype construction. Meanwhile, pathogenic variants/SNPs of target genes were amplified at our hospital, followed by Sanger sequencing (Shanghai Sangon). In one cycle of case 4, specific short tandem repeat (STR) markers were detected via capillary electrophoresis using an ABI 3130 XL genetic analyzer (Applied Biosystems) to validate the genotype of embryos. Primer sequences for variants and markers are shown in Table S1. PCR was performed using the GoTaq Master Mix 2× kit (Promega) as per the manufacturer's instructions. The genotype of embryos was determined based on the results of pathogenic variants and haplotype analysis (Harton et al., 2011).

### 2.6 Blastocyst transfer and clinical outcome

The disease‐free embryos were transferred, and pregnancy outcomes were followed up as previously reported (Tan et al., 2014). To verify the results of PGT‐M, amniocentesis was performed at 17–18 weeks of gestation, and genomic DNA was extracted from amniocytes for prenatal diagnosis.

## 3 RESULTS

A total of four couples were enrolled in this study according to the recruitment criteria. Mosaic pathogenic variants were detected via ultra‐deep sequencing, and the recurrence risk of monogenic diseases was successfully eliminated by NGS‐based PGT‐M.

### 3.1 Identification of somatic mosaicism

**FIGURE 1:**
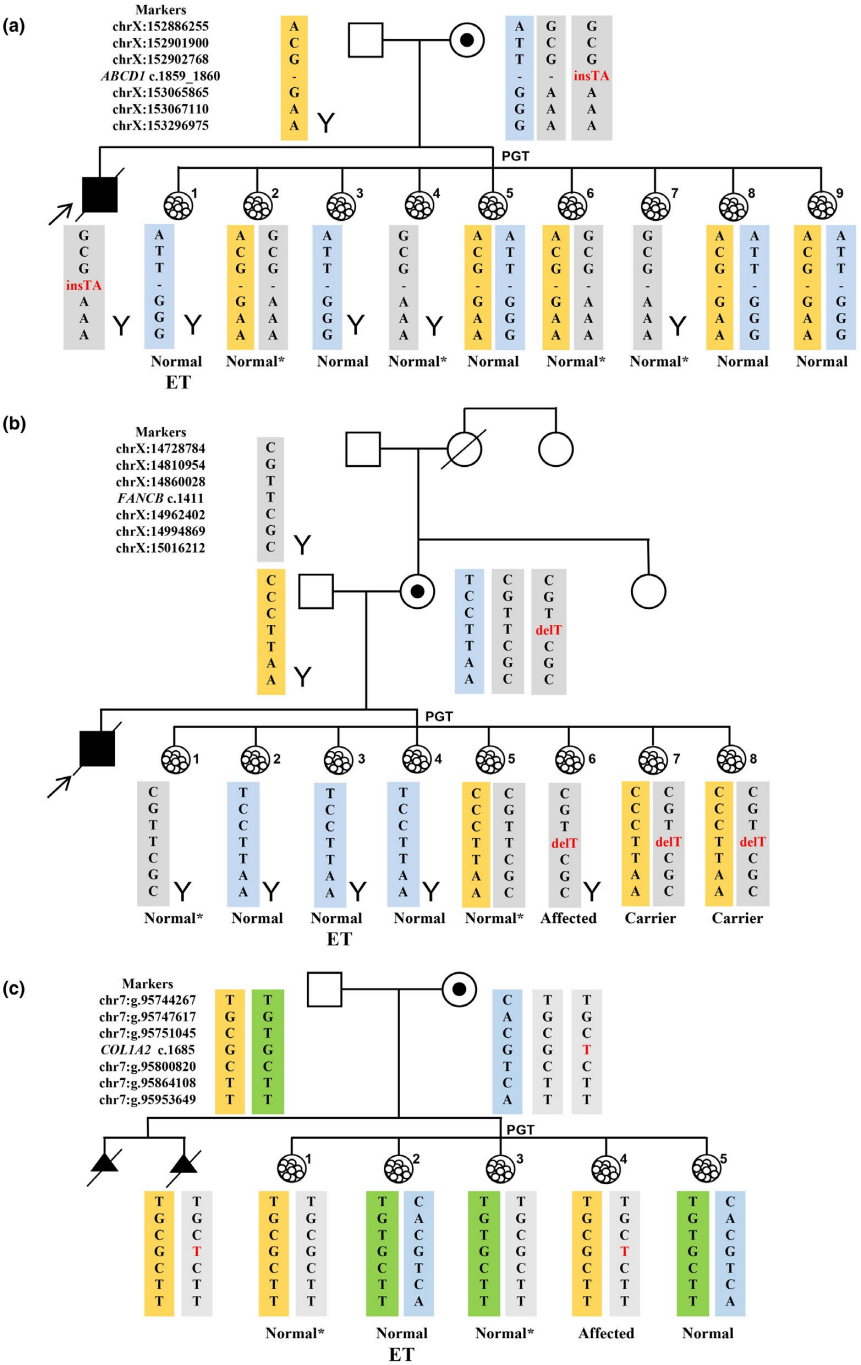
The haplotypes of family members and blastocysts of cases 1–3. The yellow and green rectangles indicate the paternal haplotype, the blue rectangles indicate the maternal wild‐type haplotype, and the gray rectangles indicate the maternal disease‐causing haplotype with variant and the disease‐causing haplotype without variant. (a) Case 1, X‐linked adrenoleukodystrophy. Embryos 1, 3, 5, 8, and 9 inherited the wild‐type maternal haplotype. Embryos 2, 4, 6, and 7 inherited the disease‐causing maternal haplotype with no variant. Embryo 1 was transplanted. (b) Case 2, X‐linked Fanconi anemia. Embryos 2, 3, and 4 inherited the wild‐type maternal haplotype; embryos 1 and 5 inherited the disease‐causing haplotype without variant; embryos 6, 7, and 8 inherited the disease‐causing haplotype with the variant. Embryo 3 was transplanted. (c) Case 3, Autosomal dominant skeletal malformations. Embryos 2 and 5 inherited the wild‐type maternal haplotype; embryos 1 and 3 inherited the disease‐causing haplotype without variant; embryo 4 inherited the disease‐causing haplotype with variant. Embryo 2 was transplanted. *Embryos with disease‐causing haplotypes without variant. ET, embryo transplantation.

Four couples were enrolled in this study according to the recruitment criteria. Couple 1 (case 1) gave birth to a boy with X‐linked adrenoleukodystrophy (Figure 1a). Sanger sequencing of the *ABCD1* gene (NM_000033.3) showed that the patient carried the c.1859_1860insTA (p. His621Thrfs*16) variant, whereas the corresponding altered allele signal of his mother was very low (Figure S1a). Couple 2 (case 2) gave birth to a boy with Fanconi anemia who died (Figure 1b). Sanger sequencing showed that the female partner was heterozygous for c.1411delT(p. Ser471Glnfs*4) variant in *FANCB* (NM_001018113.1) (Figure S1b), which may be a *de novo* pathogenic variant, as the mutate allele signal was low in the female, and this alteration was absent in her father, aunt, and sister. Maternal somatic mosaicism was indicated by the low mutant allele signal at these variant sites via Sanger sequencing. Thereafter, ultra‐deep sequencing revealed that the proportions of the mutant cells were 9.0% and 27.6% in the women from cases 1 and 2, respectively (Table 1).

**TABLE 1:** Four cases of female *de novo* sequence variants with mosaicism.

Monogenic disease	Case 1	Case 2	Case 3	Case4
	X‐linked adrenoleukodystrophy	Fanconi anemia	Skeletal malformations	Neurofibromatosis
Female age (year)	33	37	33	31
Mode of inheritance	XR	XR	AD	AD
Gene	*ABCD1*	*FANCB*	*COL1A2*	*NF1*
Sequence variants	NM_000033.3: c.1859_1860insTA (p. His621 Thrfs*16)	NM_001018113.1: c.1411delT (p. Ser471Glnfs*4)	NM_000089.3:c.1685G>T (p. Gly562Val)	NM_000267.3:entire *NF1* gene deletion
Novel/Previously reported	Novel	Novel	Previously reported	Previously reported
Phenotype of the female	Unaffected	Unaffected	Unaffected	Affected
Sample Type, Proportion of sequence variant cells %	Blood, 9.0	Blood, 27.60	Blood, 1.12	Blood, 91.03; Oral mucosa cells, 15.70
Read depth at variant position	5462X	5129X	3573X	2580X; 1002X
No. of Affected children/fetuses	1(died)	1(died)	2 (abortion)	No pregnancy

The female of couple 3 (case 3) experienced two consecutive pregnancy terminations, as the ultrasound showed the two fetuses had skeletal malformations (Figure 1c). The second aborted fetus was subjected to genetic analysis, and a c.1685G>T(p.Gly562Val) heterozygous variant in *COL1A2* (NM_000089.3) was identified. However, the couple lacked this pathogenic variant, as assessed by Sanger sequencing. Ultra‐deep sequencing revealed that the female exhibited mosaicism for the *COL1A2* variant, in a proportion of 1.12% (Table 1).

**FIGURE 2:**
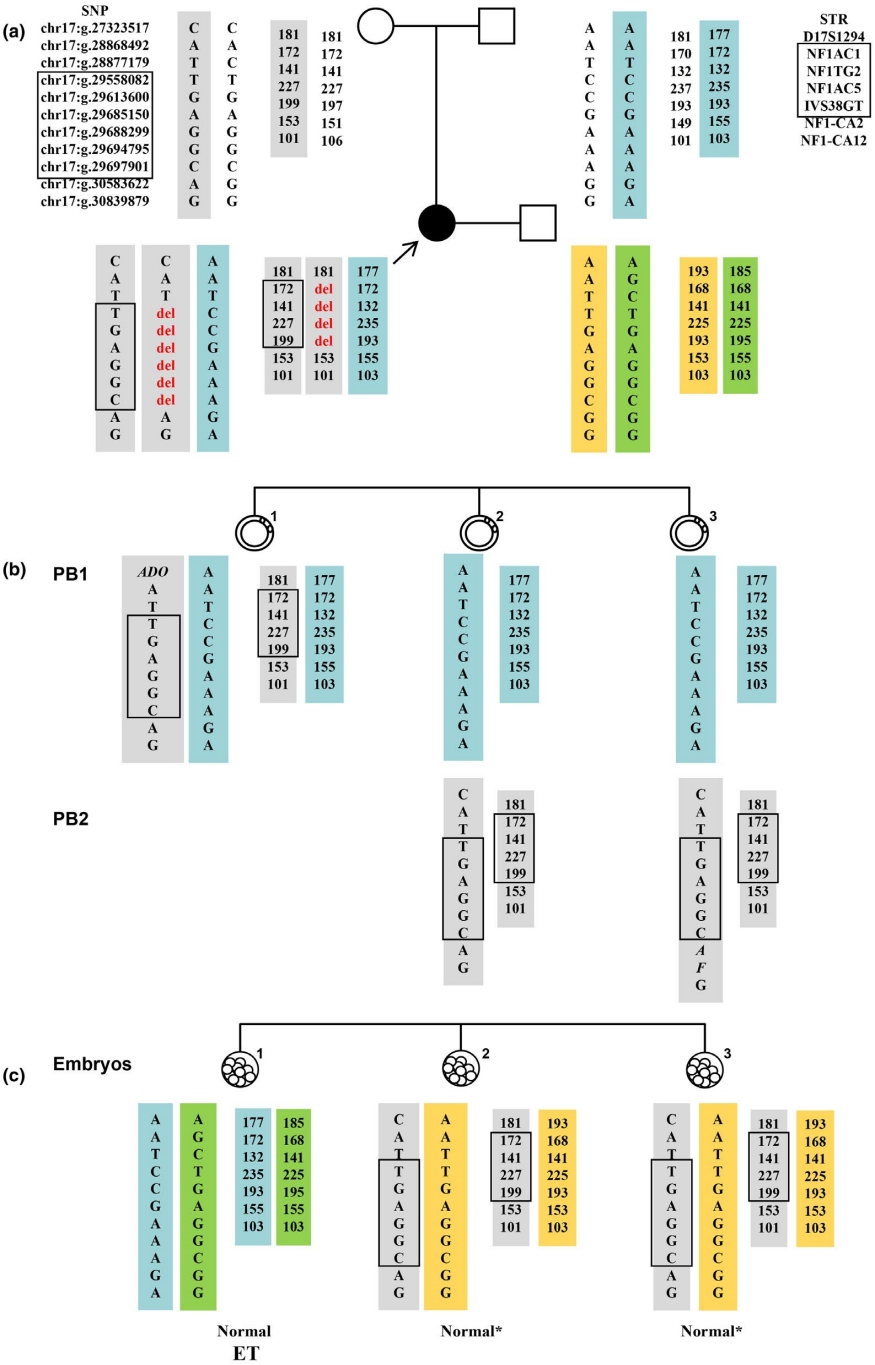
The haplotypes of family members, polar bodies, and blastocysts in case 4. The yellow and green rectangles indicate the paternal haplotype, the blue rectangles indicate the maternal wild‐type haplotype, and the gray rectangles indicate the maternal disease‐causing haplotype with variant and the disease‐causing haplotype without variant. The black box indicates the deletion area. (a) The haplotypes of family members. The female was a neurofibromatosis patient resulting from a *de novo* deletion variant. The *de novo* deletion occurred on the patient's maternal chromosome. (b) The haplotypes of polar bodies. PB2 of embryo 1 was degraded. None of the polar bodies carried the deletion. (c) The haplotypes of blastocysts. None of the three blastocysts carried the deletion; the heterozygous STR signals in the *NF1* gene of embryos 2 and 3 were direct evidence that these embryos did not carry the deletion variant. Embryo 1 was transferred. *embryos with disease‐causing haplotypes without variant; ET, embryo transplantation; PB, polar body.

In couple 4 (case 4), the female partner was affected with neurofibroma, but her parents were healthy (Figure 2a). MLPA assay revealed that she carried a heterozygous deletion of the entire *NF1* gene (NM_000267.3). As none of the other family members carried this deletion, we inferred that it was a *de novo* pathogenic variant. The SNP haplotypes of relatives revealed that the *de novo* deletion had occurred in the proband's maternal allele. Interestingly, a set of 18 SNPs in the deletion regions of the proband showed a low proportion of heterozygosity (Table S2), and the average sequencing depth of paternal and maternal signals was 2364× and 215×, respectively. These results indicated that the sequence variant exhibited mosaic status in the peripheral blood of the patient. Six of the eighteen SNPs were present in the SNP haplotype (indicated by a black box in Figure 2a). According to earlier reported methods of calculation (Biesecker & Spinner, 2013; Summerer et al., 2019), most cells of the patient carried the deletion (91.03%), whereas a small percentage of cells (8.97%) did not exhibit this sequence variant in the blood (Table S2). In addition, MLPA analysis was performed on the proband's DNA extracted from oral mucosal cells and showed that the *NF1* copy number was normal. However, analysis of the 18 SNPs using the MiSeq Sequencing System showed that only a small proportion of oral mucosal cells (15.7%) carried the deletion (Table S3).

### 3.2 Outcome of PGT‐M

PGT‐M was successfully implemented, a total of 25 blastocysts from the four couples were biopsied and 5 PBs were biopsied in one cycle. The haplotype of every embryo/polar body was successfully determined using the NGS platform and described as wild‐type haplotype, disease‐causing haplotype with variant, and disease‐causing haplotype with no variant (Table 2).

**TABLE 2:** PGT‐M outcomes of four female *de novo* sequence variants with mosaicism.

Monogenic disease	Case1	Case2	Case3	Case4
	X‐linked adrenoleukodystrophy	Fanconi anemia	Skeletal malformations	Neurofibromatosis
Oocytes obtained	16	17	15	4
Oocytes fertilized	13	15	8	3
No. of blastocysts	9	8	5	3
Polar body biopsy	NO	NO	NO	YES
PGT‐M result	5	3	2	1
No. of embryos with wild‐type haplotype				
No. of embryos with disease‐causing haplotype with variant	0	3	1	0
No. of embryos with disease‐causing haplotype but no variant	4	2	2	2
Embryo transplantation	1 Wild‐type embryo	1 Wild‐type embryo	1 Wild‐type embryo	1 Wild‐type embryo
Prenatal diagnosis	YES	YES	YES	YES
Pregnancy outcome	A healthy boy was born	A healthy boy was born	A healthy girl was born	A healthy boy was born

In case 1 (Figure 1a), nine blastocysts were biopsied and no pathogenic variants were detected. Embryos 1, 3, 5, 8, and 9 inherited the wild‐type maternal haplotype. Embryos 2, 4, 6, and 7 inherited the disease‐causing maternal haplotype without the *ABCD1* variant. Embryo 1 was transferred; this was confirmed by prenatal diagnosis, and a healthy boy was born.

In case 2 (Figure 1b), a total of eight blastocysts were biopsied. The haplotype analysis revealed that embryos 2, 3, and 4 inherited the wild‐type maternal haplotype; embryos 1 and 5 inherited the disease‐causing haplotype without the *FANCB* variant, and embryos 6, 7, and 8 inherited the disease‐causing haplotype with the variant. The haplotype information of embryos 6, 7, and 8 suggested that the c.1411delT variant in the *FANCB* gene of the female was a *de novo* mutation that occurred in the paternal chromosome. Embryo 3 was transplanted successfully; this was confirmed by prenatal diagnosis and resulted in the birth of a healthy boy.

In case 3 (Figure 1c), five blastocysts were biopsied in the PGT cycle, and the results showed that embryos 2 and 5 had inherited wild‐type maternal haplotype. The other three embryos inherited the same maternal chromosome as the second aborted fetus; however, the *COL1A2* variant was not detected in embryos 1 and 3. This couple decided to transfer embryo 2, resulting in a singleton pregnancy. Prenatal diagnosis showed consistent results with PGT, and a healthy girl was born.

In case 4 (Figure 2b,c), three blastocysts and the corresponding PBs were biopsied (PB2 of embryo 1 was degraded). The SNP haplotype indicated that obvious heterozygous SNP signals existed in the *NF1* gene of embryo 1 PB1. The PB1 of embryos 2 and 3 inherited the wild‐type haplotype, whereas PB2 inherited the disease‐causing haplotype lacking *NF1* deletion. Embryo 1 inherited the wide‐type maternal haplotype. Embryos 2 and 3 inherited the disease‐causing haplotype but did not carry the deletion based on the analysis of PBs. To verify the results, STR haplotypes were constructed. The heterozygous STR signals in the *NF1* gene of embryos 2 and 3 were direct evidence that these embryos did not carry the pathogenic variant. Therefore, all three embryos were normal and transplantable. Prenatal diagnosis confirmed that the female proband received embryo 1, and a healthy boy was born.

## 4 DISCUSSION

In this study, ultra‐deep sequencing was used to confirm that the four women harbored mosaic pathogenic variants in four different genes, and NGS‐based PGT‐M was employed to reduce the recurrence risk of monogenic diseases. During four PGT cycles, trophectoderm cells from 25 blastocysts and 5 PBs from three oocytes were subjected to genetic testing. A wild‐type embryo was transferred for each woman and confirmed by prenatal diagnosis, resulting in the birth of four healthy infants. This is the first report that illustrates the successful application of NGS‐based PGT‐M to prevent the transmission of maternal mosaic pathogenic variants to offspring.

Our study showed that ultra‐deep sequencing was an effective and sensitive method to identify mosaic sequence variants. In our study, Sanger sequencing showed that the women included in cases 1 and 2 carried heterozygous variants in *ABCD1* and *FANCB*, respectively, and the signal intensity of the altered allele was very low. Additionally, Sanger sequencing confirmed that the woman in case 3 did not exhibit the *COL1A2* variant. Furthermore, MLPA revealed that the woman in case 4 was a carrier of a heterozygous deletion in the *NF1* gene. However, the four women exhibited mosaicism, confirmed by ultra‐deep sequencing, with the mosaicism proportion ranging from 1.12% to 91.03% for the mutant cells (Table 1). Our findings suggested that even exceptionally low proportions of mosaic variants can be identified via ultra‐deep sequencing techniques, which was in agreement with previous studies (Campbell et al., 2014; Gajecka, 2016; Stosser et al., 2018).

It should be considered as a mosaic carrier in a couple who have a child with Mendelian genetic diseases due to *de novo* pathogenic variants. Subsequently, whether the parent is mosaicism should be confirmed, and the objective assessment of the recurrence risk could be provided. In the past clinical practice, asymptomatic parents delivering a child with a dominant genetic disorder is usually explained by the offspring carrying *de novo* pathogenic variant. After the birth of a second child with the same disorder, the existence of parental mosaicism is inferred (Altarescu et al., 2012). In our study, the couple in case 3 experienced two pregnancies wherein the two fetuses received a diagnosis of osteogenesis imperfecta. This indicated parental mosaicism, which was further confirmed by ultra‐deep sequencing. Thus, mosaic parents carry a higher risk of giving birth to affected offspring than the general population. In contrast, the risk of recurrence among mosaic carriers was lower than that of heterozygous carriers; this lightened the patient's psychological burden, raising their confidence to give birth to a healthy child.

The level of mosaicism in different tissues may exhibit surprising variability, even within the same embryonic lineage (Goriely et al., 2010). Our results supported the observation that even if a very low proportion of altered alleles is found in peripheral blood, a higher percentage of sequence variants may exist in other tissues. In our study, the peripheral blood of the woman in case 3 exhibited mosaicism for the *COL1A2* variant at a low rate (1.12%). However, the mosaicism may have been predominant in germ cells, as the woman had two consecutively affected fetuses. Additionally, the proportion of mutant cells was as high as 91.03% in the peripheral blood of the woman from case 4, but only 15.70% in her oral mucosal cells and all tested embryos did not exhibit the variant. Therefore, it is reasonable to conduct PGT‐M for individuals with mosaicism to avoid the transmission of genetic disorders.

The strategy of pathogenic variants detection combined with SNP haplotype analysis may be more suitable for PGT‐M in mosaic individuals. In our study, the SNP haplotype analysis showed that the male embryos 4 and 7 inherited the disease‐causing chromosome in case 1, indicating that these embryos may be affected. However, the variant detection analysis showed that the two embryos did not exhibit *ABCD1* alteration, suggesting that these embryos were actually healthy. Similarly, embryo 1 of case 2 was judged to be a normal male. When the results of pathogenic variants conflict with haplotypes in PGT of X‐linked disorder, the genotype of male embryos (with only one X chromosome) is preferred to depend on the results of pathogenic variants detection. In the same situation, female embryos (with two X chromosomes) have the risk of misdiagnosis due to allele drop out (ADO). However, when wild‐type embryos are not available, such female embryos can be considered to transfer after the couples’ understanding and accepting the risks. The detection of pathogenic variants is essential for PGT‐M in parental mosaicism to lower false‐positive results and increases the number of embryos for transfer (ESHRE PGT‐M Working Group et al., 2020).

Our study highlighted the importance of PB information in the determination of embryo genotypes for maternal mosaicism. In our study, although the five embryos (embryos 2 and 6 in case 1, embryo 5 in case 2, and embryos 1 and 3 in case 3) did not exhibit the pathogenic variants, they were suggested not to be a priority for transplantation due to the fact that ADO could not be ruled out completely. The genetic analysis of trophectoderm cell and PB could define the definite genotype of embryos, and avoid misdiagnosis due to ADO in sequence variants detection. The embryos 2 and 3 of case 4 inherited the “disease‐causing chromosomes,” but these two embryos were definitely diagnosed as a normal condition, combining with the SNP information of PBs. Therefore, PB analysis eliminates the wastage of normal embryos in PGT‐M for maternal mosaicism.

## 5 CONCLUSION

Herein, we demonstrated the validity of ultra‐deep sequencing in the identification of mosaicism and the necessity of NGS‐based PGT for the detection of single‐gene disorders with mosaicism. NGS‐based PGT is an effective strategy for the detection of monogenic diseases resulting from mosaicism. Furthermore, NGS‐based PGT combined with PB genetic analysis can improve the accuracy of PGT for monogenic diseases due to maternal mosaicism. Large‐scale studies are needed to further evaluate the efficacy of this approach. This approach will assist clinicians to undertake a more objective assessment of disease recurrence risk for mosaic individuals and obtain the accurate diagnosis of the genotype of embryos for reproductive interventions.

## CONFLICT OF INTEREST

The authors declare that they have no conflict of interest.

## AUTHORS CONTRIBUTIONS

XH, GXL, GL, and JD conceived and designed the study. XH, WBH, JD, YZ, and ZXW carried out the experiments and analyzed the data. SPZ, KLL, and FG provided the clinical samples. XH, WL, and JD interpreted the data. XH, WBH, SMY, and YQT wrote the manuscript. JD critically commented on and edited the manuscript. All authors read and approved the final version of the manuscript.

## Supporting information

Fig S1

Table S1

Table S2

Table S3

## Data Availability

The data that support the findings of this study are available on request from the corresponding author. The data are not publicly available due to privacy or ethical restrictions.

## References

[mgg31662-bib-0001] Altarescu, G. , Beeri, R. , Eldar‐Geva, T. , Varshaver, I. , Margalioth, E. J. , Levy‐Lahad, E. , & Renbaum, P. (2012). PGD for germline mosaicism. Reproductive Biomedicine Online, 25(4), 390–395. 10.1016/j.rbmo.2012.07.003 22884613

[mgg31662-bib-0002] Biesecker, L. G. , & Spinner, N. B. (2013). A genomic view of mosaicism and human disease. Nature Reviews Genetics, 14(5), 307–320. 10.1038/nrg3424 23594909

[mgg31662-bib-0003] Campbell, I. M. , Shaw, C. A. , Stankiewicz, P. , & Lupski, J. R. (2015). Somatic mosaicism: implications for disease and transmission genetics. Trends in Genetics, 31(7), 382–392. 10.1016/j.tig.2015.03.013 25910407 PMC4490042

[mgg31662-bib-0004] Campbell, I. M. , Yuan, B. O. , Robberecht, C. , Pfundt, R. , Szafranski, P. , McEntagart, M. E. , Nagamani, S. C. S. , Erez, A. , Bartnik, M. , Wiśniowiecka‐Kowalnik, B. , Plunkett, K. S. , Pursley, A. N. , Kang, S.‐H. , Bi, W. , Lalani, S. R. , Bacino, C. A. , Vast, M. , Marks, K. , Patton, M. , … Stankiewicz, P. (2014). Parental somatic mosaicism is underrecognized and influences recurrence risk of genomic disorders. American Journal of Human Genetics, 95(2), 173–182. 10.1016/j.ajhg.2014.07.003 25087610 PMC4129404

[mgg31662-bib-0005] ESHRE PGT‐M Working Group , Carvalho, F. , Moutou, C. , Dimitriadou, E. , Dreesen, J. , Giménez, C. , Goossens, V. , Kakourou, G. , Vermeulen, N. , Zuccarello, D. , & De Rycke, M. (2020). ESHRE PGT Consortium good practice recommendations for the detection of monogenic disorders. Human Reproduction Open, 2020(3), hoaa018. 10.1093/hropen/hoaa018 32500103 PMC7257022

[mgg31662-bib-0006] Gajecka, M. (2016). Unrevealed mosaicism in the next‐generation sequencing era. Molecular Genetics and Genomics, 291(2), 513–530. 10.1007/s00438-015-1130-7. 26481646 PMC4819561

[mgg31662-bib-0007] Goriely, A. , Lord, H. , Lim, J. , Johnson, D. , Lester, T. , Firth, H. V. , & Wilkie, A. O. (2010). Germline and somatic mosaicism for FGFR2 mutation in the mother of a child with Crouzon syndrome: Implications for genetic testing in "paternal age‐effect" syndromes. American Journal of Medical Genetics. Part A, 152A(8), 2067–2073. 10.1002/ajmg.a.33513 20635358 PMC2988406

[mgg31662-bib-0008] Handyside, A. H. , Harton, G. L. , Mariani, B. , Thornhill, A. R. , Affara, N. , Shaw, M. A. , & Griffin, D. K. (2010). Karyomapping: a universal method for genome wide analysis of genetic disease based on mapping crossovers between parental haplotypes. Journal of Medical Genetics, 47(10), 651–658. 10.1136/jmg.2009.069971 19858130

[mgg31662-bib-0009] Harton, G. L. , De Rycke, M. , Fiorentino, F. , Moutou, C. , SenGupta, S. , Traeger‐Synodinos, J. , & Harper, J. C. (2011). ESHRE PGD consortium best practice guidelines for amplification‐based PGD. Human Reproduction, 26(1), 33–40. 10.1093/humrep/deq231 20966462

[mgg31662-bib-0010] Ihle, M. A. , Fassunke, J. , König, K. , Grünewald, I. , Schlaak, M. , Kreuzberg, N. , Tietze, L. , Schildhaus, H. U. , Büttner, R. , & Merkelbach‐Bruse, S. (2014). Comparison of high resolution melting analysis, pyrosequencing, next generation sequencing and immunohistochemistry to conventional Sanger sequencing for the detection of p. V600E and non‐p.V600E BRAF mutations. BMC Cancer, 14, 13. 10.1186/1471-2407-14-13 24410877 PMC3893431

[mgg31662-bib-0011] Jamuar, S. S. , Lam, A.‐T. , Kircher, M. , D’Gama, A. M. , Wang, J. , Barry, B. J. , Zhang, X. , Hill, R. S. , Partlow, J. N. , Rozzo, A. , Servattalab, S. , Mehta, B. K. , Topcu, M. , Amrom, D. , Andermann, E. , Dan, B. , Parrini, E. , Guerrini, R. , Scheffer, I. E. , … Walsh, C. A. (2014). Somatic mutations in cerebral cortical malformations. The New England Journal of Medicine, 371(8), 733–743. 10.1056/NEJMoa1314432. 25140959 PMC4274952

[mgg31662-bib-0012] Khosravi, S. , Salehi, M. , Ramezanzadeh, M. , Mirzaei, H. , & Salehi, R. (2016). Novel multiplex fluorescent PCR‐based method for HLA typing and preimplantational genetic diagnosis of β‐thalassemia. Archives of Medical Research, 47(4), 293–298. 10.1016/j.arcmed.2016.07.006 27664489

[mgg31662-bib-0013] Konstantinidis, M. , Prates, R. , Goodall, N. N. , Fischer, J. , Tecson, V. , Lemma, T. , Chu, B. , Jordan, A. , Armenti, E. , Wells, D. , & Munné, S. (2015). Live births following Karyomapping of human blastocysts: experience from clinical application of the method. Reproductive Biomedicine Online, 31(3), 394–403. 10.1016/j.rbmo.2015.05.018 26206283

[mgg31662-bib-0014] Levin, I. , Almog, B. , Shwartz, T. , Gold, V. , Ben‐Yosef, D. , Shaubi, M. , Amit, A. , & Malcov, M. (2012). Effects of laser polar‐body biopsy on embryo quality. Fertility and Sterility, 97(5), 1085–1088. 10.1016/j.fertnstert.2012.02.008 22365340

[mgg31662-bib-0015] Malcov, M. , Reches, A. , Ben‐Yosef, D. , Cohen, T. , Amit, A. , Dgany, O. , Tamary, H. , & Yaron, Y. (2010). Resolving a genetic paradox throughout preimplantation genetic diagnosis for autosomal dominant severe congenital neutropenia. Prenatal Diagnosis, 30(3), 207–211. 10.1002/pd.2437 20049848

[mgg31662-bib-0016] Miyatake, S. , Koshimizu, E. , Hayashi, Y. K. , Miya, K. , Shiina, M. , Nakashima, M. , Tsurusaki, Y. , Miyake, N. , Saitsu, H. , Ogata, K. , Nishino, I. , & Matsumoto, N. (2014). Deep sequencing detects very‐low‐grade somatic mosaicism in the unaffected mother of siblings with nemaline myopathy. Neuromuscular Disorders, 24(7), 642–647. 10.1016/j.nmd.2014.04.002 24852243

[mgg31662-bib-0017] Naja, R. P. , Dhanjal, S. , Doshi, A. , Serhal, P. , Delhanty, J. , & SenGupta, S. B. (2016). The impact of mosaicism in preimplantation genetic diagnosis (PGD): approaches to PGD for dominant disorders in couples without family history. Prenatal Diagnosis, 36(9), 864–870. 10.1002/pd.4874 27441947

[mgg31662-bib-0018] Patel, B. , Byrne, J. , Phillips, A. , Hotaling, J. M. , & Johnstone, E. B. (2018). When standard genetic testing does not solve the mystery: a rare case of preimplantation genetic diagnosis for campomelic dysplasia in the setting of parental mosaicism. Fertility and Sterility, 110(4), 732–736. 10.1016/j.fertnstert.2018.05.002 30196970

[mgg31662-bib-0019] Rechitsky, S. , Pomerantseva, E. , Pakhalchuk, T. , Pauling, D. , Verlinsky, O. , & Kuliev, A. (2011). First systematic experience of preimplantation genetic diagnosis for de‐novo mutations. Reproductive Biomedicine Online, 22(4), 350–361. 10.1016/j.rbmo.2011.01.005 21324748

[mgg31662-bib-0020] Steffann, J. , Michot, C. , Borghese, R. , Baptista‐Fernandes, M. , Monnot, S. , Bonnefont, J. P. , & Munnich, A. (2014). Parental mosaicism is a pitfall in preimplantation genetic diagnosis of dominant disorders. European Journal of Human Genetics, 22(5), 711–712. 10.1038/ejhg.2013.164 24022303 PMC3992558

[mgg31662-bib-0021] Stosser, M. B. , Lindy, A. S. , Butler, E. , Retterer, K. , Piccirillo‐Stosser, C. M. , Richard, G. , & McKnight, D. A. (2018). High frequency of mosaic pathogenic variants in genes causing epilepsy‐related neurodevelopmental disorders. Genetics in Medicine, 20(4), 403–410. 10.1038/gim.2017.114 28837158 PMC5895461

[mgg31662-bib-0022] Summerer, A. , Schäfer, E. , Mautner, V. F. , Messiaen, L. , Cooper, D. N. , & Kehrer‐Sawatzki, H. (2019). Ultra‐deep amplicon sequencing indicates absence of low‐grade mosaicism with normal cells in patients with type‐1 NF1 deletions. Human Genetics, 138(1), 73–81. 10.1007/s00439-018-1961-5 30478644

[mgg31662-bib-0023] Tan, Y. , Yin, X. , Zhang, S. , Jiang, H. , Tan, K. E. , Li, J. , Xiong, B. O. , Gong, F. , Zhang, C. , Pan, X. , Chen, F. , Chen, S. , Gong, C. , Lu, C. , Luo, K. , Gu, Y. , Zhang, X. , Wang, W. , Xu, X. , … Lin, G. E. (2014). Clinical outcome of preimplantation genetic diagnosis and screening using next generation sequencing. GigaScience, 3(1), 30. 10.1186/2047-217X-3-30 25685330 PMC4326468

[mgg31662-bib-0024] Tarilonte, M. , Morín, M. , Ramos, P. , Galdós, M. , Blanco‐Kelly, F. , Villaverde, C. , Rey‐Zamora, D. , Rebolleda, G. , Muñoz‐Negrete, F. J. , Tahsin‐Swafiri, S. , Gener, B. , Moreno‐Pelayo, M. A. , Ayuso, C. , Villamar, M. , & Corton, M. (2018). Parental Mosaicism in PAX6 Causes Intra‐Familial Variability: Implications for Genetic Counseling of Congenital Aniridia and Microphthalmia. Frontiers in Genetics, 9, 479. 10.3389/fgene.2018.00479 30386378 PMC6199369

[mgg31662-bib-0025] Viart, V. , Willems, M. , Ishmukhametova, A. , Dufernez, F. , Anahory, T. , Hamamah, S. , Schmitt, S. , Claustres, M. , & Girardet, A. (2017). Germline mosaicism is a pitfall in PGD for X‐linked disorders. Single sperm typing detects very low frequency paternal gonadal mosaicism in a case of recurrent chondrodysplasia punctata misattributed to a maternal origin. Prenatal Diagnosis, 37(2), 201–205. 10.1002/pd.4982 27943351

[mgg31662-bib-0026] Wright, C. F. , Prigmore, E. , Rajan, D. , Handsaker, J. , McRae, J. , Kaplanis, J. , Fitzgerald, T. W. , FitzPatrick, D. R. , Firth, H. V. , & Hurles, M. E. (2019). Clinically‐relevant postzygotic mosaicism in parents and children with developmental disorders in trio exome sequencing data. Nature Communications, 10(1), 2985. 10.1038/s41467-019-11059-2 PMC6611863 31278258

