# Supplementary figures and images for: Next‐generation sequence‐based preimplantation genetic testing for monogenic disease resulting from maternal mosaicism
Source: Mol Genet Genomic Med. 2021 May 4;9(5):e1662. doi: 10.1002/mgg3.1662 (PMC8172198; doi:10.1002/mgg3.1662)

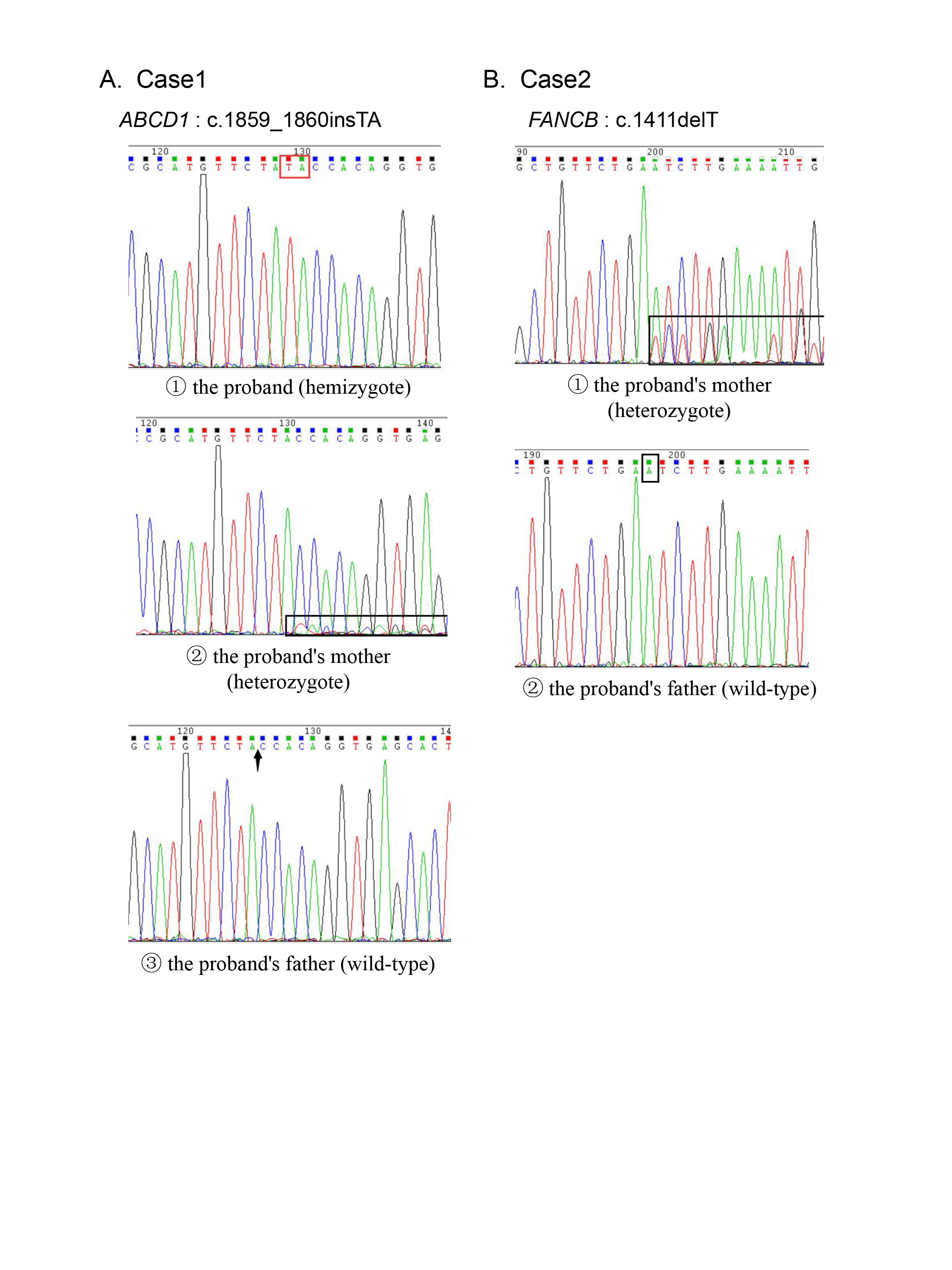

Supplement: Supplementary file 1 — Fig S1 [file MGG3-9-e1662-s003.jpg]
