# Supplementary material for: Next‐generation sequence‐based preimplantation genetic testing for monogenic disease resulting from maternal mosaicism
Source: Mol Genet Genomic Med. 2021 May 4;9(5):e1662. doi: 10.1002/mgg3.1662 (PMC8172198; doi:10.1002/mgg3.1662)
Supplement: Supplementary file 2 — Table S1 [file MGG3-9-e1662-s004.doc]

Supplementary Table 1. Primer sequences for pathogenic variants/markers.

| Gene | Pathogenic variants/Markers | Primer(5'-3') | | Fluorescence labeled |
| --- | --- | --- | --- | --- |
| *ABCD1（NM_000033.3）* | c.1859_1860insTA | F | GTCACAGCTAGCTCATTCCCG | - |
|  |  | R | GGCTCAGGCTCCACTGAG | - |
| *FANCB* (NM_001018113.1) | c.1411delT | F | CCATAAGCCCTAGCCAAAAG | - |
|  |  | R | CAGTAATAATAATTTCCCTTGCTTC | - |
| *COL1A2* (NM_000089.3) | c.1685G>T | F | CCTGAGGCTTTGAGACAT | - |
|  |  | R | ATTTGGCTCATTCTCTCC | - |
| *NF1* (NM_000267.3) | rs6505234 | F | CAACCATCCATAGACCATCCTGA | - |
|  |  | R | ATGAATAGCACCTCCAGGGTC | - |
|  | D17S1294 | F | GAGGTTGAGCCTGCAATAAG | 5’6-FAM |
|  |  | R | TTCTTTCCTTACTAAGTTGAGAACG | - |
|  | NF1AC1 | F | TGTCCTCAAGTGATCGGC | 5’6-FAM |
|  |  | R | CGATCAACTGACACATCCAT | - |
|  | NF1TG2 | F | TCCTGTTTTTAGTGAGTGCGTA | 5’6-FAM |
|  |  | R | TCATGCCACTGCACTCTAG | - |
|  | NF1AC5 | F | AATGTAACAATTGTGGAACTGC | 5’HEX |
|  |  | R | GCTGGGATTACAGGTGTGAG | - |
|  | IVS38GT | F | CAACAGAGCAAGACCCTGTCTC | 5’HEX |
|  |  | R | CCATTAGCACCCCTCCTAACAT | - |
|  | NF1-CA2 | F | GGTTGGGTTGAAATCTCTGG | 5’6-FAM |
|  |  | R | TTTCAGTAGCCTTCCTCCTG | - |
|  | NF1-CA12 | F | CCTACAACTGCCAACCTTTG | 5’6-FAM |
|  |  | R | TCCCTGGATCCATAGAGAAGT | - |
