# Supplementary material for: Next‐generation sequence‐based preimplantation genetic testing for monogenic disease resulting from maternal mosaicism
Source: Mol Genet Genomic Med. 2021 May 4;9(5):e1662. doi: 10.1002/mgg3.1662 (PMC8172198; doi:10.1002/mgg3.1662)
Supplement: Supplementary file 3 — Table S2 [file MGG3-9-e1662-s001.doc]

Supplementary Table 2

The proportion of normal cells in the peripheral blood of the proband in case 4 with *NF1* deletion as determined by PGT-M based on NGS. Totally, heterozygosity was observed at 18 SNPs located within the *NF1* deletion region. Listed are the proportions of alleles identified as determined by the number of sequence reads covering the respective SNPs. The proband’s maternal allele represents with the lower allele count.

| SNP | Genomic position (hg19) | Maternal allele | | | Paternal allele | | | Proportion of normal cells^†^ |
| --- | --- | --- | --- | --- | --- | --- | --- | --- |
|  |  | Base | Reads  (×) | Proportion  (%) | Base | Reads  (×) | Proportion  (%) |  |
| 1 | 29558082 | T | 586 | 8.38 | C | 6405 | 91.62 | 9.15 |
| 2 | 29560777 | T | 104 | 8.31 | G | 1148 | 91.69 | 9.06 |
| 3 | 29567152 | T | 65 | 9.04 | C | 654 | 90.96 | 9.94 |
| 4 | 29570587 | G | 166 | 7.33 | T | 2100 | 92.67 | 7.9 |
| 5 | 29578724 | C | 259 | 9.29 | T | 2528 | 90.71 | 10.25 |
| 6 | 29580882 | G | 420 | 9.60 | A | 3953 | 90.4 | 10.62 |
| 7 | 29587917 | A | 43 | 7.05 | C | 567 | 92.95 | 7.58 |
| 8 | 29613600 | G | 270 | 7.18 | C | 3488 | 92.82 | 7.74 |
| 9 | 29627297 | G | 127 | 7.79 | C | 1503 | 92.21 | 8.45 |
| 10 | 29685150 | A | 271 | 7.78 | G | 3213 | 92.22 | 8.43 |
| 11 | 29688299 | G | 271 | 9.08 | A | 2714 | 90.92 | 9.99 |
| 12 | 29691368 | C | 105 | 7.90 | A | 1224 | 92.1 | 8.58 |
| 13 | 29694795 | G | 189 | 8.39 | A | 2065 | 91.61 | 9.15 |
| 14 | 29697901 | C | 509 | 7.95 | A | 5893 | 92.05 | 8.64 |
| 15 | 29699859 | C | 180 | 9.01 | G | 1817 | 90.99 | 9.91 |
| 16 | 29699860 | T | 181 | 9.08 | A | 1812 | 90.92 | 9.99 |
| 17 | 29703438 | C | 69 | 8.53 | G | 740 | 91.47 | 9.32 |
| 18 | 29735829 | A | 50 | 6.37 | G | 735 | 93.63 | 6.80 |
|  |  | Mean | 215 | 8.23 |  | 2364 | 91.77 | 8.97 |

^†^: The proportion of normal cells was calculated by means of the formula:

N=(M/P)×100%

N: Proportion of normal cells

M: Number of maternal alleles

P: Number of Paternal alleles
