# Supplementary material for: Next‐generation sequence‐based preimplantation genetic testing for monogenic disease resulting from maternal mosaicism
Source: Mol Genet Genomic Med. 2021 May 4;9(5):e1662. doi: 10.1002/mgg3.1662 (PMC8172198; doi:10.1002/mgg3.1662)
Supplement: Supplementary file 4 — Table S3 [file MGG3-9-e1662-s002.doc]

Supplementary Table 3

The proportion of normal cells in the oral mucosa cells of the proband in case 4 with *NF1* deletion as determined by PGT-M based on NGS. In total, heterozygosity was observed at 18 SNPs located within the *NF1* deletion region. Listed are the proportions of alleles identified as determined by the number of sequence reads covering each SNPs. The proband’s maternal allele represents with the lower allele count.

| SNP | Genomic position (hg19) | Maternal allele | | | Paternal allele | | | Proportion of normal cells^†^ |
| --- | --- | --- | --- | --- | --- | --- | --- | --- |
|  |  | Base | Reads  (×) | Proportion  (%) | Base | Reads  (×) | Proportion  (%) |  |
| 1 | 29558082 | T | 915 | 46.24 | C | 1064 | 53.76 | 86.01 |
| 2 | 29560777 | T | 223 | 44.16 | G | 282 | 55.84 | 79.08 |
| 3 | 29567152 | T | 175 | 44.42 | C | 219 | 55.58 | 79.92 |
| 4 | 29570587 | G | 296 | 45.75 | T | 351 | 54.25 | 84.33 |
| 5 | 29578724 | C | 281 | 43.36 | T | 367 | 56.64 | 76.55 |
| 6 | 29580882 | G | 667 | 44.32 | A | 838 | 55.68 | 79.60 |
| 7 | 29587917 | A | 75 | 49.34 | C | 77 | 50.66 | 97.39 |
| 8 | 29613600 | G | 440 | 47.16 | C | 493 | 52.84 | 89.25 |
| 9 | 29627297 | G | 441 | 42.08 | C | 607 | 57.92 | 72.65 |
| 10 | 29685150 | A | 533 | 45.99 | G | 626 | 54.01 | 85.15 |
| 11 | 29688299 | G | 805 | 45.07 | A | 981 | 54.93 | 82.05 |
| 12 | 29691368 | C | 254 | 46.44 | A | 293 | 53.56 | 86.71 |
| 13 | 29694795 | G | 316 | 46.88 | A | 358 | 53.12 | 88.25 |
| 14 | 29697901 | C | 1739 | 43.36 | A | 2272 | 56.64 | 76.55 |
| 15 | 29699859 | C | 90 | 47.12 | G | 101 | 52.88 | 89.11 |
| 16 | 29699860 | T | 87 | 46.03 | A | 102 | 53.97 | 85.29 |
| 17 | 29703438 | C | 243 | 46.02 | G | 285 | 53.98 | 85.25 |
| 18 | 29735829 | A | 549 | 48.54 | G | 582 | 51.46 | 94.33 |
|  |  | Mean | 452 | 45.68 |  | 550 | 54.32 | 84.30 |

^†^: The proportion of normal cells was calculated by means of the formula:

N=(M/P)×100%

N: Proportion of normal cells

M: Number of maternal alleles

P: Number of Paternal alleles
